# Discriminating between Light- and Heavy-Tailed Distributions with Limit Theorem

**DOI:** 10.1371/journal.pone.0145604

**Published:** 2015-12-23

**Authors:** Krzysztof Burnecki, Agnieszka Wylomanska, Aleksei Chechkin

**Affiliations:** 1 Hugo Steinhaus Center, Department of Mathematics, Wroclaw University of Technology, Wroclaw, Poland; 2 Akhiezer Institute for Theoretical Physics, National Science Center “Kharkov Institute of Physics and Technology”, Kharkov, Ukraine; 3 Max-Planck Institute for Physics of Complex Systems, Dresden, Germany; 4 Institute for Physics and Astronomy, University of Potsdam, Potsdam, Germany; Indiana University Bloomington, UNITED STATES

## Abstract

In this paper we propose an algorithm to distinguish between light- and heavy-tailed probability laws underlying random datasets. The idea of the algorithm, which is visual and easy to implement, is to check whether the underlying law belongs to the domain of attraction of the Gaussian or non-Gaussian stable distribution by examining its rate of convergence. The method allows to discriminate between stable and various non-stable distributions. The test allows to differentiate between distributions, which appear the same according to standard Kolmogorov–Smirnov test. In particular, it helps to distinguish between stable and Student’s *t* probability laws as well as between the stable and tempered stable, the cases which are considered in the literature as very cumbersome. Finally, we illustrate the procedure on plasma data to identify cases with so-called L-H transition.

## Introduction

The Central Limit Theorem which we know from the university courses on probability theory teaches us that the distributions of sums of independent random variables having finite variance converge to a Gaussian distribution [1]. This fundamental property of the Gaussian probability law explains its prevalence in all branches of science dealing with randomness. However, it is also a well-known fact that the heavy-tailed probability distributions with diverging variance are ubiquitous in nature and finance [2–6]. Among them the alpha-stable (Lévy) distributions first investigated by Paul Lévy [7] possess a remarkable place. Due to the *Generalized* Central Limit Theorem they attract distributions of sums of random variables with diverging variance, similarly to the Gaussian law that attracts distributions with finite variance [8]. Thus, as the Gaussian law, the Lévy stable laws naturally appear when evolution of a system or result of an experiment are determined by a sum of random factors.

The Lévy stable probability densities have the asymptotics decaying at infinity as |*x*|^−1−*α*^, where *α* is the index of stability, or the Lévy index, varying between 0 and 2. They attract distributions having the same law of decay. On the contrary, the Gaussian distribution has the Lévy index 2 and attracts all distributions with lighter tails, i.e. decaying faster than 1/|*x*|^3^ (“normal” convergence to a normal law [8]).

Lévy statistics may appear asymptotically due to the Generalized Central Limit Theorem like, for example, in non-Brownian continuous time random walks with jumps and/or waiting times obeying heavy-tailed distributions, see the reviews [9–12]. In many problems the appearance of the heavy-tailed distributions and limiting Lévy statistics can be well-understood theoretically, like, e.g., in the famous Holzmark problem [13], diffusion of photons in hot atomic vapours [14], light propagation in fractal medium called Lévy glass [15], “paradoxical” particle diffusion on a fast-folding polymer [16, 17], and motion of tracer particles in a dilute suspension of swimmers [18]. On the other hand, in many complex systems the conclusion about heavy tail existence is based solely on empirical data analysis, since reliable theoretical models explaining such an existence do not exist. Among such we mention fluctuation processes characterized by bursts or large outliers that are inherent to many phenomena far from equilibrium. Thus, stably distributed random noises are observed in such diverse applications as plasma turbulence (density and electric field fluctuations [19–22]), stochastic climate dynamics [23, 24], physiology (heartbeats [25]), electrical engineering [26], biology [27], and economics [28]. Heavy-tailed distributions govern circulation of dollar bills [29] and behavior of the marine verterbrates in response to patchy distribution of food resources [30].

In view of developing theoretical models for such complex phenomena, it is of vital importance to have a reliable tool to distinguish between light- and heavy-tailed probability distributions in empirical data analysis. In the literature different methods are proposed, the most popular are Kolmogorov–Smirnov, Cramer–von-Mises and Anderson–Darling tests [31]. Though those tests are commonly used in different applications, there is a strong evidence available that they are capable of detecting only a very limited range of alternatives [32]. In the literature one can also find other statistical methods used for the two-sample problem, like the data-driven rank test introduced in [33] which exploits the core of Neyman’s testing approach [34] based on the popular likelihood ratio method. The idea of Neyman was also extended by many authors in different applications, see, e.g. [35]. In [2] an extensive study of twenty-four real-world datasets from a range of different disciplines was performed in order to check how convincing the power-law model is as a fit in comparison to other distributions, but the results for the log-normal and stretched exponential distributions were quite ambiguous.

We would also like to mention methods specifically tailored to testing for the Gaussian and Lévy distributions. They rely on concrete properties of these distributions, like, e.g., measures of the moments for the Gaussian case [36, 37] or summation property of the Lévy stable distribution [38]. A problem of recognizing *α*-stable Lévy distribution with Lévy index close to 2 from experimental data was addressed in [22].

In this paper we suggest another algorithm to distinguish between light- and heavy-tailed probability laws. The idea of the algorithm, which is visual and easy to implement, is to check whether the underlying law belongs to the domain of attraction of the Gaussian or non-Gaussian stable distribution by examining its rate of convergence. Below in section Results we demonstrate by different examples, that our algorithm is more efficient than the standard Kolmogorov-Smirnov test. We note that the limit theorems which specify the rate of convergence to the stable law in one and multi-dimensional cases have been studied in [39–41]. The problem of convergence to a stable distribution via order statistics was considered in [42]. The general aspects related to convergence to the stable laws were addressed in [43].

Before we proceed to the description of the algorithm suggested, it is worthwhile to dwell on empirical observations which inspired us to tackle the problem of discriminating between light- and heavy-tailed distributions.

### Plasma turbulence in fusion devices

It is well known that the edge plasma turbulence, which is characterized by the high level of fluctuations of the charged particle density and electrical field, plays a decisive role in generation of anomalous particle and heat fluxes from the plasma confinement region in various types of closed magnetic confinement systems, see, e.g., [44, 45] and the literature cited therein. Knowing fluctuation statistics is vital for constructing theoretical model of the phenomenon. There are different statements in plasma literature on this subject. In [19, 21] the authors report about the Lévy stable statistics of the edge plasma fluctuations, whereas in [20] the truncated Lévy distribution is observed. In [46] for the description of bursty transport in plasma turbulence the stochastic process was suggested which is a squared Gaussian process (“square Gaussian distribution”). In Section Results we demonstrate how our algorithm discriminates between the Lévy stable and truncated Lévy distributions, and between the Lévy stable and square Gaussian ones. We also address the issue of changing fluctuation statistics at the so-called L-H transition.

### Search strategies, biological movements

It is generally believed that random search processes based on scale free Lévy stable jump length distributions optimize the search for sparse targets [47]. Lévy flights and Lévy walks, i.e. random walks with scale-free jump length distributions were indeed shown to optimize the search for sparse targets as supported by extensive movement data of many animal species and humans. However, in several cases the reports of Lévy statistics are debated [48–51]. Moreover, whereas a reanalysis of albatross flights showed that they generally do not obey Lévy statistics [52], strong evidence was presented according to which Lévy flights are indeed a search pattern for individuals [53].

### Monitoring machine’s condition

The problem of recognition between distributions for two vectors of observations appears also in the engineering for the machine’s condition monitoring issue. We can mention here the analysis of condition of planetary gearboxes in the time-variable operating conditions [54], where the authors found different properties of signals registered for a machine in a good and bad condition. The discrimination between distributions for signals of healthy and unhealthy machines can be a starting point to a condition diagnostics. This issue was also considered in [55].

### Modeling asset returns

The theoretical rationale for modeling asset returns by the Gaussian distribution comes from the Central Limit Theorem. This has been notoriously contradicted with empirical findings [56]. The fact that non-Gaussian Lévy stable distributions are leptokurtic and can accommodate fat tails and asymmetry, has led to their use as an alternative model for asset returns since the 1960s. Mandelbrot’s seminal work [57] on applying stable distributions in finance gained support in the first few years after its publication, but subsequent works have questioned the stable distribution hypothesis, in particular, the stability under summation (for a review see [58]). Several authors have found a very good agreement of high-frequency returns with a stable distribution up to six standard deviations away from the mean [59]. For more extreme observations, however, the distribution they found fall off approximately exponentially. To cope with such observations the so-called truncated Lévy distributions were introduced in [60]. The original definition postulated a sharp truncation of the stable probability density function at some arbitrary point. Later, however, exponential smoothing was proposed in [61].

## Materials and Methods

### Rate of convergence to a stable law

We consider the normalized sum of *n* continuous independent identically distributed (i.i.d.) random variables *X*_*i*_, *i* = 1, …, *n* with the common cumulative distribution function *F*(*x*) and probability density function *f*(*y*) = *F*′(*y*).

Following [41] we recall some basic results about the convergence of the normalized sums

$$\begin{array}{c} Y_{n}=\frac{1}{B_{n}}\sum_{i=1}^{n}\left(X_{i}-A_{n}\right). \end{array}$$ (1)

When *F* has first and second moments *m*_1_ and *m*_2_, we set *B*_*n*_ = *n* ^1/2^ and *A*_*n*_ = *m*_1_ and by the Central Limit Theorem, as *n* → ∞, the distribution of *Y*_*n*_ converges to a normal distribution with mean zero and second moment *m*_2_. If *F* has a third moment *m*_3_, then *f*_*n*_(*y*) − *f*(*y*) = *O*(*n* ^−1/2^), where *f*_*n*_(*y*) is the density function of *Y*_*n*_. This is the case of considered here non-symmetric tempered stable and square Gaussian distributions. If *m*_3_ = 0, then the rate of convergence is *o*(*n* ^−1/2^). This condition is true for considered here symmetric tempered stable and Student’s *t* distributions. However, if *F* does not have a third moment, but if *F*(*x*) = *O*(|*x*|^*α*^) as *x* → −∞ and *F*(*x*) = 1 − *O*(*x* ^*α*^) as *x* → ∞ with 2 < *α* ≤ 3, then *f*_*n*_(*y*) − *f*(*y*) = *O*(*n* ^−(*α*−2)/2^). In this case we see that the rate of convergence is slower than when there is a third moment.

When *F* does not have both first and second moments, the distribution of the *Y*_*n*_ may still converge. A necessary and sufficient condition for this is

$$\begin{array}{c} F\left(x\right)=\{\begin{array}{cc} \left(c_{1}+r_{1}\left(x\right)\right){\left|x\right|}^{-\alpha } & \text{if}\;x<0,\\ \left(c_{2}+r_{2}\left(x\right)\right){\left|x\right|}^{-\alpha } & \text{if}\;x>0, \end{array} \end{array}$$

with 0 < *α* ≤ 2, *c*_1_ and *c*_2_ positive constants, *r*_1_(*x*)→0 as *x* → −∞ and *r*_2_(*x*)→0 as *x* → ∞. When this condition holds and 0 < *α* < 2 we can set *B*_*n*_ = *n* ^1/*α*^ in Eq (1) and by the Generalized Central Limit Theorem the limit is a stable distribution. In [41] one can find convergence rates for different symmetric and non-symmetric densities. The results are illustrated by means of Monte Carlo simulations.

All recalled here basic results on the convergence to a stable distribution are given as the function of the difference between density of the aggregated (and normalized) random variables and that of the limiting distribution. Different rates of convergence to a stable law can be also studied in a similar way with the use of distribution functions, and also characteristic functions. In section Results we analyze rate of convergence of the estimated index of stability of the aggregated samples to that of a limiting distribution via the regression method which is equivalent to the characteristic function approach.

### One- and two-sample Kolmogorov-Smirnov tests

To test whether a dataset follows a specific distribution (one sample test) one can apply a general testing procedure based on empirical distribution function (EDF) [22, 62–64] or to employ specific properties of the distribution [22, 37].

We consider here only continuous random variables. Let us concentrate on EDF tests. A statistics measuring the difference between the empirical *F*_*n*_(*x*) and the analytical *F*(*x*) distribution function, called an EDF statistic, is based on the vertical difference between the distributions. This distance is usually measured either by a supremum or a quadratic norm [62–64].

The most popular supremum statistic:

$$\begin{array}{c} D=\underset{x}{\sup}|F_{n}\left(x\right)-F\left(x\right)|, \end{array}$$ (2)

is known as the Kolmogorov or Kolmogorov-Smirnov (KS) statistic.

This statistic can be applied to perform a rigorous statistical test. The null hypothesis is that a specific distribution is acceptable, whereas the alternative is that it is not. Small values of its test statistic *D* are evidence in favor of the hypothesis, large ones indicate its falsity [62]. To see how unlikely such a large outcome would be if the hypothesis is true, we calculate the *p*-value by: *p*-value = *P*(*D* ≥ *t*), where *t* is the statistic value for a given sample. It is typical to reject the hypothesis when a small *p*-value is obtained, like, e.g., below 1%, 3% or 5%. To calculate *p*-values for the EDF tests one can apply the procedure proposed in [65] and described in detail in [64].

To employ any of the tests, first, we need to estimate the parameters of the hypothetical distribution. In the Gaussian case the standard method is the maximum likelihood. In the non-Gaussian stable case we use the fast and accurate regression method [66–68]. The method is based on characteristic function which is given in a simple form for a non-Gaussian stable distribution in contrast to its probability density function.

Now, let us turn to two-sample tests. Tests based on EDF may be generalized to allow comparison of the distributions of the two datasets. The two sample Kolmogorov–Smirnov test is quite standard and is implemented in many mathematical packages like, e.g., Matlab (we used the function “kstests2”), R, Octave or Statistica. In this case, the Kolmogorov–Smirnov statistic is

$$\begin{array}{c} D2=\underset{x}{\sup}|F_{1,n}\left(x\right)-F_{2,n}\left(x\right)|, \end{array}$$ (3)

where *F*_1,*n*_ and *F*_2,*n*_ are the empirical distribution functions of the first and the second sample respectively.

Note that the two-sample test checks whether the two data samples come from the same distribution. This does not specify the common distribution (e.g. Gaussian or non-Gaussian stable).

### Discrimination algorithm

We consider two samples of observations of length *N*: {*x*_1_, *x*_2_, …, *x*_*N*_} and {*y*_1_, *y*_2_, …, *y*_*N*_}. The main idea of this algorithm is to distinguish between the domains of attraction of different underlying distributions. The classic result of probability theory states that a normalized sum of arbitrary i.i.d. random variables converges, if at all, to an *α*-stable (*α* < = 2) random variable [69]. The convergence holds in distribution and its rate varies from distribution to distribution [41].

We apply this fact in the following testing procedure. In this procedure we test whether both datasets belong to the same domain of attraction, either of the Gaussian law (finite second moment, light-tailed case) or of the non-Gaussian stable law (infinite second moment, heavy-tailed case).

1. We divide the dataset into non-overlapping consecutive blocks of length *K* = 1, 2, …, 10. Next, we sum the values within each block and obtain aggregated data of length [*N*/*K*] (*K* = 1 refers the whole dataset). Finally, we estimate the index of stability *α* for the constructed data via the regression method [66–68].
2. We plot the estimated index of stability with respect to *K* = 1, 2, …, 10.
  - If the estimated values converge to 2, then the data are light-tailed and belong to the domain of attraction of the Gaussian law. In particular, if the data are Gaussian, the estimated values should be equal to 2 for most of the cases.
  - If the estimated values converge to *α* < 2, then the data are heavy-tailed and belong to the domain of attraction of the non-Gaussian stable law. In particular, if the data are (*α* < 2)-stable, the estimated values should be always close to *α*.

To sum up, we try to find to which domain of attraction (Gaussian or non-Gaussian stable) belongs the distribution underlying the data. This is done by aggregating the data and observing the behavior of the estimated index of stability. The estimation is performed via the regression method, hence, technically, we study the convergence of the characteristic function to that of the limiting distribution. We also note that our method is fairly simple to implement and only requires a regression method estimator, which is easily accessible for many mathematical and statistical packages.

One can enhance this procedure by calculating box plots for the estimated values *α*. This is intended to help to access if the differences in convergence are statistically justified. The box plot provides a statistical information about the distribution of the values [70]. Precisely, it produces a box and whisker plot for each value of *α*. The box has lines at the lower quartile, median, and upper quartile values. The whiskers are lines extending from each end of the box to show the extent of the rest of the data. Points are drawn as outliers if they are larger than *Q*3 + 1.5(*Q*3 − *Q*1) or smaller than *Q*1 − 1.5(*Q*3 − *Q*1), where *Q*1 and *Q*3 are lower and upper quartiles, respectively. This corresponds to the 99.3% coverage if the data are normally distributed. The plotted whisker extends to the adjacent value, which is the most extreme data value that is not an outlier.

But, how to create box plots from a single dataset? The idea is to generate more samples from one sample (from its empirical distribution function). This procedure is called bootstrapping in statistics [71]. The bootstrapping is done for the whole dataset. For large datasets one can skip plotting box plots or to replace them with the plot of the mean estimated alpha values obtained via the bootstrapping procedure.

## Results

### Testing of the procedure on the simulated data

We illustrate here the quality of the discrimination algorithm introduced in section Materials and Methods. To this end we examine several light- and heavy-tailed distributions, among them Gaussian, non-Gaussian stable and non-stable. We selected cases (pairs), which are very difficult (or even impossible) to distinguish from their plots, or from their empirical cumulative distributions functions (CDFs) and probability distributions functions (PDFs). To make the pairs as close as possible, the parameters of the second distribution (i.e. stable) were chosen on the basis of the first distribution. More precisely, by using the first sample we estimated the stable distribution parameters and the obtained values were used for generation of the second sample. For each case we simulated a sample of length 2000.

#### Gaussian and non-Gaussian stable distributions

We consider here the Gaussian distribution with *μ* = 0 and $\sigma =\sqrt{2}$ and the symmetric stable distribution with parameters *α* = 1.95 and *σ* = 1. We remind the reader that the stable distributed random variable *X* with parameters *α*, *σ*, *β* and *μ* is defined through its characteristic function in the following way:

$$\begin{array}{ccc} \Phi_{X}\left(k\right) & = & E\exp\{iXk\}\\ & = & \left\{ \begin{array}{ccc} \exp\{-\sigma^{\alpha }{\left|k\right|}^{\alpha }\left(1-i\beta \text{sgn}\left(k\right)\tan\frac{\pi \alpha }{2}\right)+ik\mu \} & \text{if} & \alpha \neq 1,\\ \exp\{-\sigma |k|(1-i\beta \frac{2}{\pi }\text{sgn}\left(k\right)\ln|k|)+ik\mu )\} & \text{if} & \alpha =1, \end{array}\right. \end{array}$$ (4)

where 0 < *α* ≤ 2 is the index of stability, *σ* > 0 is the scale parameter, −1 ≤ *β* ≤ 1 is the skewness parameter and *μ* ∈ *R* is the location parameter. In the case of *μ* = 0 and *β* = 0 the stable distributed random variable *X* is symmetric.

**Fig 1:**
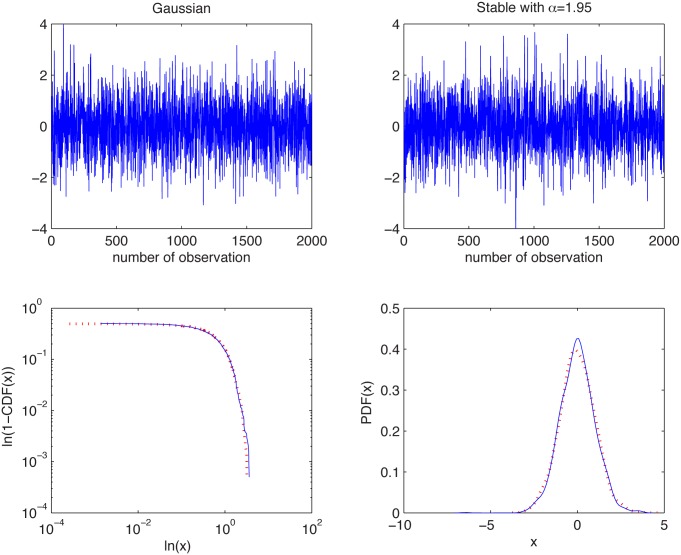
Simulated samples from the Gaussian distribution with *μ* = 0 and $\sigma =\sqrt{2}$ (top left panel) and the symmetric stable distribution with *α* = 1.95 and *σ* = 1 (top right panel), and their empirical tails (1-CDFs) in log-log scale (bottom left panel) and PDFs (bottom right panel).

**Fig 2:**
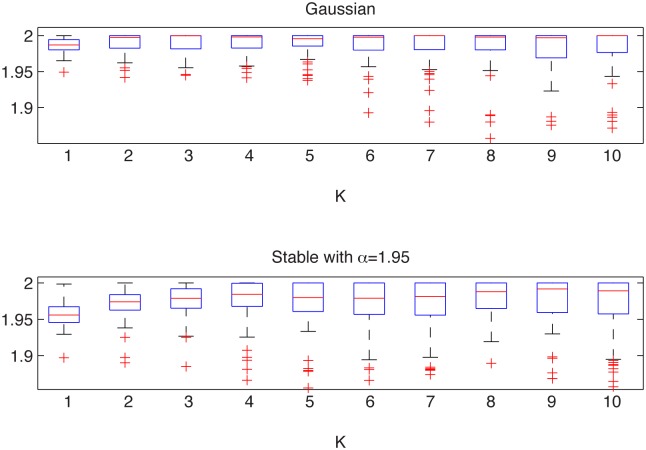
Estimated *α* values for the Gaussian sample from the top left panel of Fig 1 (top panel) and for the symmetric non-Gaussian stable sample from the top right panel of Fig 1 (bottom panel). The boxplots were constructed from 100 bootstrap samples of length 2000.

In Fig 1 we present the simulated samples and in Fig 2 we illustrate the results of the algorithm. We can observe that the estimated *α* values behave differently for the two distributions. For the Gaussian sample they are equal to 2 for most of the cases, whereas for the non-Gaussian stable sample the stability index is always smaller than 2. For both distributions the estimated values are almost independent of *K* as the aggregation does not change the index of stability. Moreover, boxplots are getting wider with increasing *K* as the estimation is performed for smaller samples, hence the variance of the estimator increases.

This leads to the conclusion that the examined distributions are different. In contrast to this, the two-sample Kolmogorov-Smirnov test (applied to normalized samples) does not reject the null hypothesis of common distributions, with *p*-value equal to 0.44, which is very high.

#### Tempered stable and stable distributions

We consider here the symmetric tempered stable distribution with parameters *α* = 1.9 and *λ* = 0.1 and the symmetric stable distribution with *α* = 1.9 and *σ* = 1. Let us mention that the tempered stable distribution was introduced in [60] and developed later in [61]. A general mathematical description of this class of distributions (and processes) was presented in [72]. In our paper we consider the tempered stable distribution with the Lévy triplet (*κ* ^2^, *ν*, *γ*), defined as follows [73]:

$$\begin{array}{ccc} \kappa & = & 0\\ \nu (dx) & = & \left({\tilde{C}}_{+}e^{-\lambda_{+}x}1_{x>0}+{\tilde{C}}_{-}e^{-\lambda_{-}\left|x\right|}1_{x<0}\right)\frac{dx}{{\left|x\right|}^{\alpha +1}}\\ \gamma & = & m-\int_{|x|>1}x\nu \left(dx\right), \end{array}$$ (5)

where ${\tilde{C}}_{+},{\tilde{C}}_{-},\lambda_{+},\lambda_{-}>0$, *α* ∈ (0, 2), and *m* ∈ *R*.

For the symmetric tempered stable distribution we take *m* = 0, *λ*_+_ = *λ*_−_ = *λ* and *C*_+_ = *C*_−_ = 1. It can be shown that in this case the Fourier transform of random variable *T* takes the following form:

$$\begin{array}{ccc} \phi_{T}\left(k\right) & = & E\exp\{-iTk\}\\ & = & \exp\{[{\left(\lambda +ik\right)}^{\alpha }+{\left(\lambda -ik\right)}^{\alpha }-2\lambda^{\alpha }]\}. \end{array}$$ (6)

**Fig 3:**
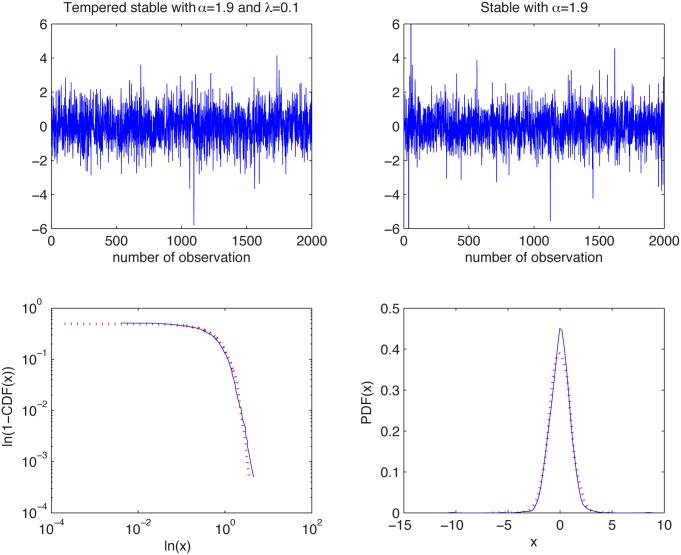
Simulated samples from the symmetric tempered stable distribution with *α* = 1.9 and *λ* = 0.1 (top left panel) and symmetric stable distribution with *α* = 1.95 and *σ* = 1 (top right panel), and their empirical tails in log-log scale (bottom left panel) and PDFs (bottom right panel).

**Fig 4:**
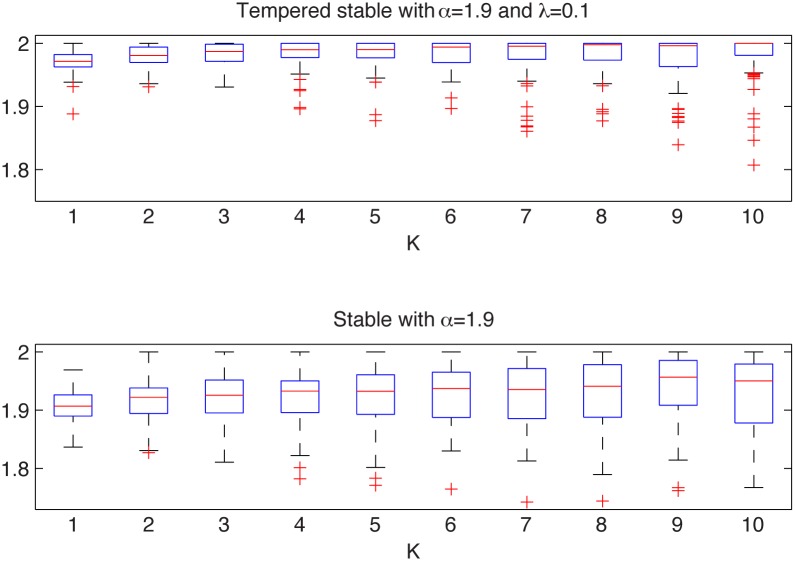
Estimated *α* values for the symmetric tempered stable sample from the top left panel of Fig 3 (top panel) and for the symmetric stable sample from the top right panel of Fig 3 (bottom panel). The box plots were constructed from 100 bootstrap samples of length 2000.

In Fig 3 we present the simulated samples and in Fig 4 we illustrate the results of the algorithm. We can observe that the estimated *α* values behave differently for the two distributions. For the tempered sample they tend to 2, whereas for the non-Gaussian stable sample the *α* values stabilize just above 1.9. For the stable distribution the estimated values are almost independent of *K* as the aggregation does not change the index of stability.

Therefore, we conclude that the analyzed distributions are different. In contrast to this, the two-sample Kolmogorov-Smirnov test does not reject the null hypothesis of common distributions, with *p*-value equal to 0.12, which is essentially greater than the significance level 5%.

#### Square Gaussian and stable distributions

The square Gaussian random variable *W* with zero-mean and unit variance is defined as follows:

$$\begin{array}{c} W=\sqrt{1-2\gamma^{2}}X+\gamma \left(X^{2}-1\right), \end{array}$$ (7)

where *X* is a standard Gaussian random variable and *γ* ^2^ ≤ 1/2. The *W* random variable is a special case of univariate non-Gaussian systems considered in [46].

**Fig 5:**
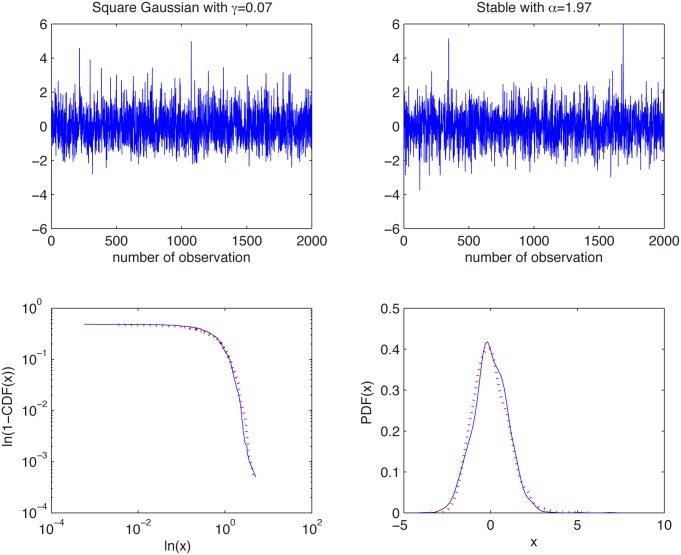
Simulated samples from the square Gaussian distribution with *γ* = 0.07 (top left panel) and stable distribution with *α* = 1.97, *β* = 1, *σ* = 0.7 and *μ* = 0.1 (top right panel), and their empirical tails in log-log scale (bottom left panel) and PDFs (bottom right panel).

**Fig 6:**
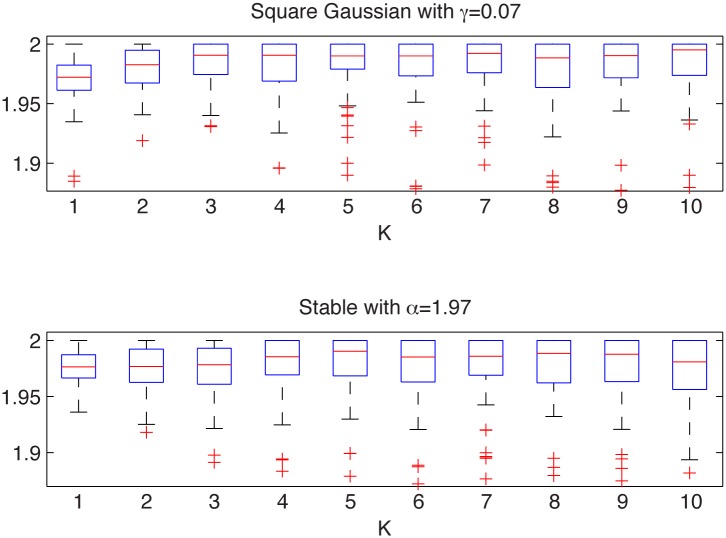
Estimated *α* values for the square Gaussian sample from the top left panel of Fig 5 (top panel) and for the stable sample from the top right panel of Fig 5 (bottom panel). The box plots were constructed from 100 bootstrap samples of length 2000.

We consider here the square Gaussian distribution with *γ* = 0.07 and the stable distribution with *α* = 1.97, *β* = 1, *σ* = 0.7 and *μ* = 0.1. In Fig 5 we present the simulated samples and in Fig 6 we illustrate the results of the algorithm. We can observe that the estimated *α* values behave differently for the two distributions. For the square Gaussian sample they tend to 2, whereas for the non-Gaussian stable sample the *α* values are below 2. For the stable distribution the estimated values are almost independent of *K* as the aggregation does not change the index of stability.

Hence, we conclude that the examined distributions are different. In contrast to this, the two-sample Kolmogorov-Smirnov test does not reject the null hypothesis of common distributions, with *p*-value equal to 0.17, which is again essentially greater than the significance level 5%.

#### Student’s *t* and stable distributions

We consider here the Student’s *t* distribution with 4 degrees of freedom and the stable distribution with *α* = 1.85, *σ* = 0.77, *β* = 0.15, and *μ* = 0.01. A random variable *Z* has the Student’s *t* distribution with *ν* degrees of freedom if it can be expressed as

$$\begin{array}{c} Z=\frac{U}{V}\sqrt{\nu }, \end{array}$$ (8)

where *U* is the standard normal random variable *N*(0, 1), *V* has *χ* ^2^-distribution with *ν* degrees of freedom and *U* and *V* are independent. The probability density function of the random variable *Z* is given by:

$$\begin{array}{c} f\left(x\right)=\frac{\Gamma (\frac{\nu +1}{2})}{\sqrt{\pi \nu }\Gamma (\frac{\nu }{2})}{\left(1+\frac{x^{2}}{\nu }\right)}^{-\frac{\nu +1}{2}},\;x\in R. \end{array}$$ (9)

**Fig 7:**
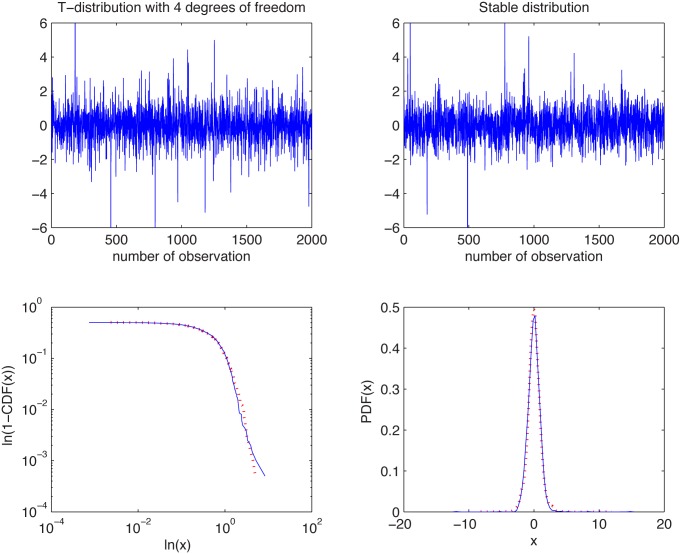
Simulated samples from the Student’s *t* distribution with 4 degrees of freedom (top left panel) and stable distribution with *α* = 1.85, *σ* = 0.77, *β* = 0.15, and *μ* = 0.01 (top right panel), and their empirical tails in log-log scale (bottom left panel) and PDFs (bottom right panel).

**Fig 8:**
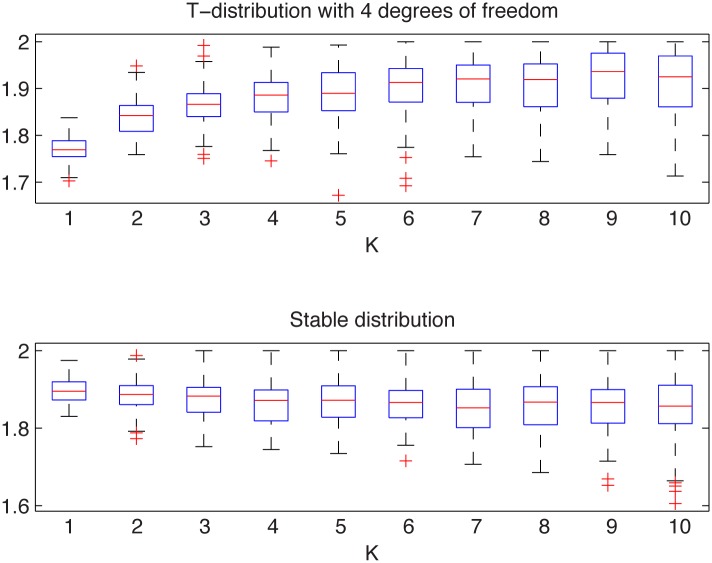
Estimated *α* values for the Student’s *t* sample from the top left panel of Fig 7 (top panel) and for the stable sample from the top right panel of Fig 7 (bottom panel). The box plots were constructed from 100 bootstrap samples of length 2000.

In Fig 7 we present the simulated samples and in Fig 8 we illustrate the results of the algorithm. We can observe that the estimated *α* values behave differently for the two distributions. For the Student’s *t* sample they tend to 2, whereas for the non-Gaussian stable sample the *α* values are around 1.9. For the stable distribution the estimated values are almost independent of *K* as the aggregation does not change the index of stability.

This clearly indicates that the analyzed distributions are different. In contrast to this, the two-sample Kolmogorov-Smirnov test does not reject the null hypothesis of common distributions, with *p*-value equal to 0.2, which is again essentially greater than the significance level 5%.

#### Symmetric stable distributions with different stability indices

We consider here two samples from the symmetric stable distribution with *σ* = 1 but different values of *α*, namely *α*_1_ = 1.85 and *α*_2_ = 1.9.

**Fig 9:**
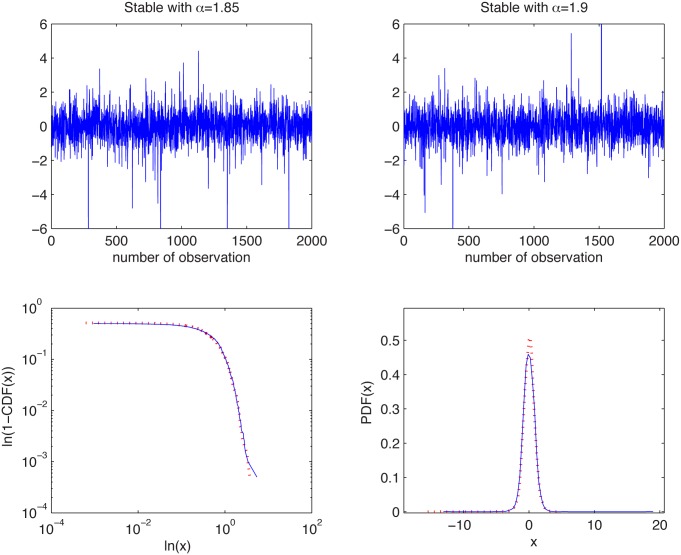
Simulated samples from the symmetric stable distribution with *α* = 1.85 and *σ* = 1 (top left panel) and symmetric stable distribution with *α* = 1.9 and *σ* = 1 (top right panel), and their empirical tails in log-log scale (bottom left panel) and PDFs (bottom right panel).

**Fig 10:**
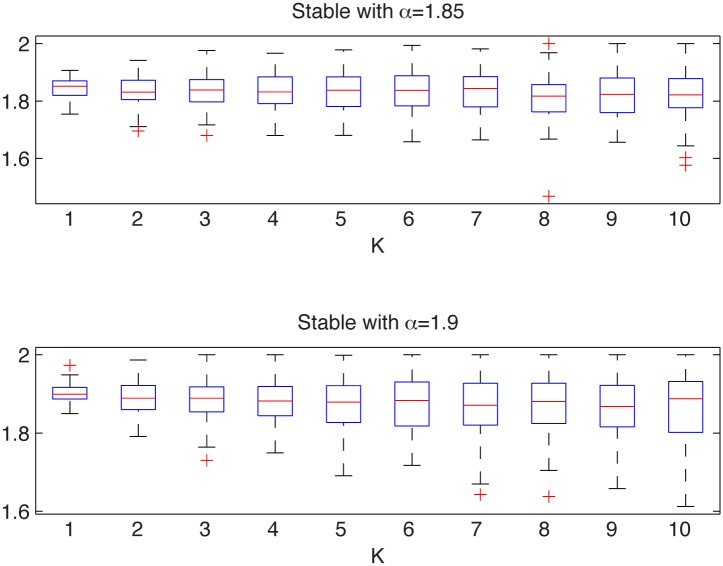
Estimated *α* values for the stable sample from the top left panel of Fig 9 (top panel) and for the stable sample from the top right panel of Fig 9 (bottom panel). The box plots were constructed from 100 bootstrap samples of length 2000.

In Fig 9 we present the simulated samples and in Fig 10 we illustrate the results of the algorithm. We can observe that the estimated *α* values behave differently for the two cases, namely they fluctuate around their true *α* values. For both cases the estimated values are almost independent of *K* as the aggregation does not change the index of stability. Finally, boxplots are getting wider with increasing *K* as the estimation is performed for smaller samples, hence the variance of the estimator increases.

Hence, we conclude that the analyzed distributions are different. In contrast to this, the two-sample Kolmogorov-Smirnov test fails, not rejecting the null hypothesis of common distributions, with *p*-value equal to 0.1, which is greater than the significance level 5%.

### Plasma data

We investigate here the data obtained in an experiment on the controlled thermonuclear fusion device. One of the most important problems related to these data is the information about statistical properties of plasma fluctuations before and after the so-called L-H transition phenomenon. This is the name of a sudden transition from the low confinement mode (L mode) to a high confinement mode (H mode) accompanied by suppression of turbulence and a rapid drop of turbulent transport at the edge of thermonuclear device [74].

We consider two datasets and want to statistically confirm if the L-H transition appeared. Precisely, we analyze floating potential fluctuations (in volts) in turbulent plasma, registered in the Uragan-3M stellarator torsatron for two torus radial positions *r* = 9.5 cm and *r* = 9.6 cm. For the detailed description of the experimental setup, see [19, 75].

#### Example 1

**Fig 11:**
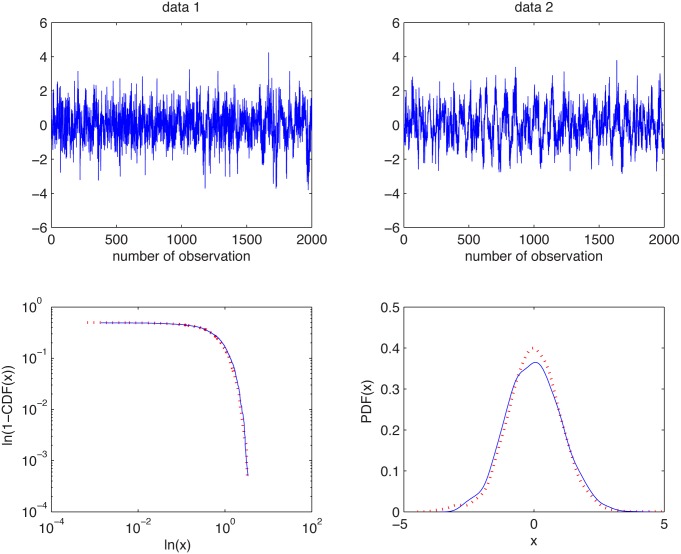
Plasma data for the torus radial position *r* = 9.5 cm: data1 (top left panel), data2 (top right panel), and their empirical tails (bottom left panel) and PDFs (bottom right panel).

The first example corresponds to the plasma data registered in the U-3M torsatron for the torus radial position *r* = 9.5 cm. We extract two subsamples from this dataset. The first subsample consists of observations with the numbers between 9000 and 11000 (data1), while the second contains observations with the numbers between 18000 and 20000 (data2). In Fig 11 we present the analyzed vectors of observations (after normalization) and the corresponding empirical tails and PDFs.

**Fig 12:**
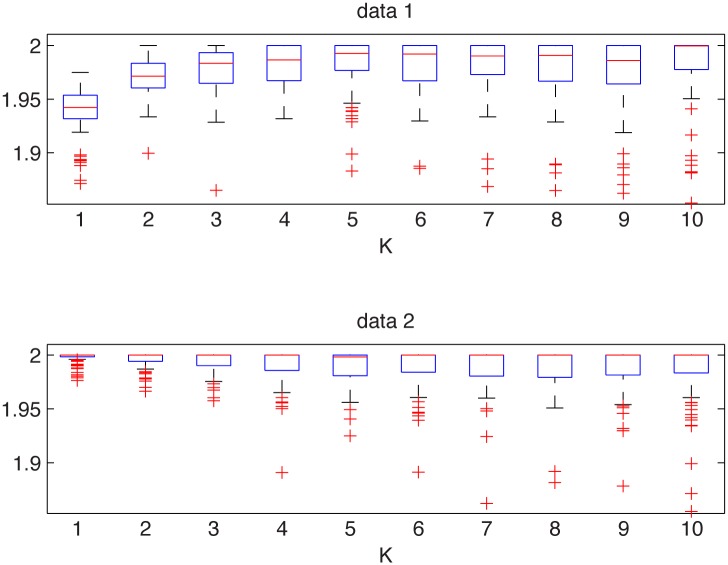
Estimated *α* values for the data1 from the top left panel of Fig 11 (top panel) and for the data2 from the top right panel of Fig 11 (bottom panel). The box plots were constructed from 100 bootstrap samples of length 2000.

In Fig 12 we depict estimated *α* values for the two subsamples. We can observe that the estimated values have a different behavior. For the first subsample, for *K* = 1, the values are essentially lower, for other *K*’s, the values increase and fluctuate around some *α* which is smaller than 2. This suggests that either the data are non-Gaussian stable or they belong to the domain of attraction of this law. For the second subsample the values lie on the line corresponding to *α* = 2. We may claim that they are Gaussian. Hence, we conclude that the underlying distributions are different. In order to confirm these results we also performed the Jarque-Bera (JB) test for Gaussianity for both subsamples [22, 27, 37]. For the data1 the test rigorously rejects the hypothesis of Gaussianity, namely the obtained *p*-value is equal to 0.001. For the data2 the *p*-value of the JB test is equal to 0.15, which confirms the data can be considered as a Gaussian sample. Moreover, we employed the Anderson-Darling (AD) test for stable distribution [22, 27, 62]. It appears that the data1 can be modeled by the non-Gaussian stable distribution. The *p*-value is equal to 0.46 and the estimated parameters of the stable distribution are: $\hat{\alpha }=1.94$, $\hat{\sigma }=3.8$, $\hat{\beta }=-0.7$ and $\hat{\mu }=-1.1$. This confirms conclusions from our test.

In contract to this, the two-sample Kolmogorov-Smirnov test does not reject the hypothesis of the same distribution, namely *p*-value is quite high and equal to 0.24.

#### Example 2

**Fig 13:**
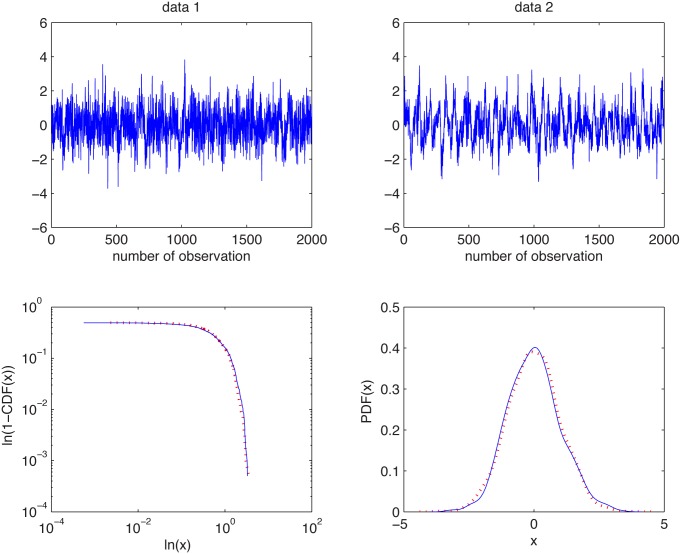
Plasma data for the torus radial position *r* = 9.6 cm: data1 (top left panel), data2 (top right panel), and their empirical tails (bottom left panel) and PDFs (bottom right panel).

The second example corresponds to the plasma data registered in the U-3M torsatron for the torus radial position *r* = 9.6 cm. We extract two subsamples from this dataset. The first subsample consists of observations with the numbers between 6000 and 8000 (data1) while the second contains observations with the numbers between 16000 and 18000 (data2). In Fig 13 we present the analyzed vectors of observations (after normalization) and the corresponding empirical tails and PDFs.

**Fig 14:**
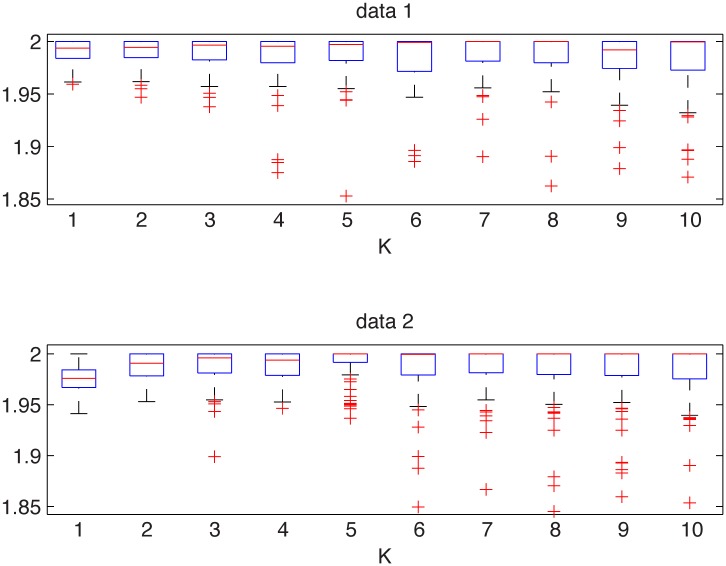
Estimated *α* values for the data1 from the top left panel of Fig 13 (top panel) and for the data2 from the top right panel of Fig 13 (bottom panel). The box plots were constructed from 100 bootstrap samples of length 2000.

In Fig 14 we depict estimated *α* values for the two subsamples. We can observe that the estimated values have different behaviour. For the first subsample the values lie on the line corresponding to *α* = 2. This suggests that the data are Gaussian. For the second subsample the values converge to 2. We may claim that they are not Gaussian but belong to the domain of attraction of Gaussian law. Hence, the conclusion is that the underlying distributions are different. In order to confirm these results we also performed the JB test for Gaussianity for both subsamples. For the data1 the test does not reject Gaussianity (the corresponding *p*-value is equal to 0.38, which is very high), whereas for data2 the Gaussianity is definitely rejected, namely the *p*-value is equal to 0.0017. Moreover, we employed the AD test for the stable distribution. It appears that the stable distribution is rigorously rejected for the data2, with the *p*-value being only 0.002. This confirms conclusions from our test.

In contract to this, the two-sample Kolmogorov-Smirnov test does not reject the hypothesis of the same distribution, namely *p*-value is extremely high and equal to 0.84.

## Discussion

In this paper we introduced an algorithm for distinguishing between light- and heavy-tailed distributions based on the generalized limit theorem. The limit theorem defines a domain of attraction of the stable law, which is different for *α* = 2 (Gaussian case) and *α* < 2 (non-Gaussian stable). In the algorithm, we divide data into non-overlapping blocks, and for the block aggregate data, we estimate the index of stability *α* via the regression method. Then, we plot the estimated values for increasing lengths of the blocks. The light-tailed case is observed as a convergence of estimated *α*’s to a Gaussian distribution (*α* = 2) (in particular, for the Gaussian data, the values should concentrate along the line *α* = 2). For the heavy-tailed case, the estimated values should converge to *α* < 2 (in particular, for the *α*-stable case, the values should concentrate along this *α*). Since in the algorithm the estimation of the index of stability is done with the help of the regression method, in fact we observe the convergence of the characteristic function of the aggregated sample to that of a limiting distribution.

The main advantages of the algorithm are:

- It is visual and very easy to implement, requires only a method of estimation of the stability index *α* (we used the regression method which is pretty standard and available for various mathematical packages)
- The method is very sensitive to differences in the tails of the underlying distributions. It is superior to the standard two-sample Kolmogorov-Smirnov case.
- It even works when the rigorous statistical tests fail, see the case of the Student’s *t* distribution.

The investigated problem has a rich and long history, spanning many fields. For example log-normal and stretched exponential distributions are often mistaken for power-law distributions [2, 76]. A problem of the indistinguishability of Student’s *t* and Lévy stable distributions was raised in [38]. We showed that our approach leads to definite conclusions for these sensitive cases.

Finally, we stress we do not pretend to claim that our test is general and suited for all cases and distributions. This is also not a rigorous test to check if the data are Gaussian or non-Gaussian stable. We propose a visual test to distinguish between two specific classes of distributions, those belonging to the domain of attraction of the Gaussian and non-Gaussian Lévy stable laws. This test also helps to differentiate between two underlying distributions belonging to the same domain of attraction but with different rate of convergence. It can be applied to many cases where classical tests fail.
